# Identification of QTLs associated with seed protein concentration in two diverse recombinant inbred line populations of pea

**DOI:** 10.3389/fpls.2024.1359117

**Published:** 2024-03-11

**Authors:** Krishna Kishore Gali, Ambuj Jha, Bunyamain Tar’an, Judith Burstin, Gregoire Aubert, Dengjin Bing, Gene Arganosa, Thomas D Warkentin

**Affiliations:** ^1^ Department of Plant Sciences, Crop Development Centre, University of Saskatchewan, Saskatoon, SK, Canada; ^2^ School of Life Sciences, Central University of Gujarat, Gandhinagar, Gujarat, India; ^3^ Agroécologie, INRAE, Institut Agro, Univ. Bourgogne, Univ. Bourgogne Franche-Comté, Dijon, France; ^4^ Agriculture and Agri-Food Canada, Lacombe, AB, Canada

**Keywords:** marker-assisted selection, *Pisum sativum*, SNP genotyping, vegetable protein, QTL

## Abstract

Improving the seed protein concentration (SPC) of pea (*Pisum sativum* L.) has turned into an important breeding objective because of the consumer demand for plant-based protein and demand from protein fractionation industries. To support the marker-assisted selection (MAS) of SPC towards accelerated breeding of improved cultivars, we have explored two diverse recombinant inbred line (RIL) populations to identify the quantitative trait loci (QTLs) associated with SPC. The two RIL populations, MP 1918 × P0540-91 (PR-30) and Ballet × Cameor (PR-31), were derived from crosses between moderate SPC × high SPC accessions. A total of 166 and 159 RILs of PR-30 and PR-31, respectively, were genotyped using an Axiom® 90K SNP array and 13.2K SNP arrays, respectively. The RILs were phenotyped in replicated trials in two and three locations of Saskatchewan, Canada in 2020 and 2021, respectively, for agronomic assessment and SPC. Using composite interval mapping, we identified three QTLs associated with SPC in PR-30 and five QTLs in PR-31, with the LOD value ranging from 3.0 to 11.0. A majority of these QTLs were unique to these populations compared to the previously known QTLs for SPC. The QTL *SPC-Ps-5.1* overlapped with the earlier reported SPC associated QTL PC-QTL-3. Three QTLs, *SPC-Ps-4.2*, *SPC-Ps-5.1*, and *SPC-Ps-7.2* with LOD scores of 7.2, 7.9, and 11.3, and which explained 14.5%, 11.6%, and 11.3% of the phenotypic variance, respectively, can be used for marker-assisted breeding to increase SPC in peas. Eight QTLs associated with the grain yield were identified with LOD scores ranging from 3.1 to 8.2. Two sets of QTLs, *SPC-Ps-2.1* and *GY-Ps-2.1*, and *SPC-Ps-5.1* and *GY-Ps-5.3*, shared the QTL/peak regions. Each set of QTLs contributed to either SPC or grain yield depending on which parent the QTL region is derived from, thus confirming that breeding for SPC should take into consideration the effects on grain yield.

## 1 Introduction

Pea (*Pisum sativum* L.) is one of the oldest domesticated legume crops (Zohary and Hopf, 1973). The global pea production in 2020 was ~14.7 million tons, of which Canada produced ~4.6 million tons (FAOSTAT, 2023). The pea crop is valued for its rich content of seed protein, fiber, vitamins, and minerals (Shanthakumar et al., 2022). Pea protein has a well-balanced amino acid profile with high content of essential amino acids lysine and threonine, high digestibility, and low allergenicity (Lu et al., 2020). However, pea seeds are low in sulfur-containing amino acids methionine and cysteine (Stone et al., 2015). The physicochemical properties of pea protein combined with its availability, affordability, and sustainable production practices make it an attractive ingredient in various food and feed applications (Shanthakumar et al., 2022; Wu et al., 2023). The use of pea protein in food products has gained immense popularity in recent years, especially among consumers looking for plant-based protein sources. Improving functionality of plant proteins will increase their usefulness as food ingredients (Akharume et al., 2021). According to a report by MarketsandMarkets Blog (2022), the global pea protein market value was estimated at USD 1.7 billion in 2022 and is projected to reach USD 2.9 billion by 2027. Increasing the SPC of grain legumes also contributes to the increasing demand for protein-rich human diets and to minimizing greenhouse gas emissions (Jha et al., 2022).

Western Canada is a major producer of peas accounting for nearly 30% of global pea production in 2020 (FAOSTAT, 2023). Pea protein processing is a growing industry in this region and provides a means of utilizing the pea crop for additional markets beyond human and animal consumption. The industry growth in this region is driven by the abundance of pea production in western Canada, increasing demand for plant-based protein ingredients, and sustainable agriculture, and adds value to the western Canadian pea crops. Peas are a low-input crop, requiring less fertilizer and pesticides than most other crops, and are known to improve soil health by fixing nitrogen for subsequent crops (Pelzer et al., 2012). This aligns with the goals of sustainable agriculture, which seeks to minimize environmental impact while maximizing production efficiency.

The average seed protein concentration (SPC) of pea is 20%–25% on a dry weight basis (Shanthakumar et al., 2022). The major constituents of seed protein in pea are albumin (10%–20%), globulin (65%–80%), prolamin, and glutelin. Pea cultivars with higher SPC are valuable for processing companies to produce a higher yield of protein per unit of raw material. The estimated commercial value of pea seeds, with high protein content based on the average retail price of pea protein isolate in the range of 25–30 USD/kg of isolate, has led to an increased focus on plant breeding programs that aim to develop pea cultivars with higher SPC. In the last decade, the pea breeding program at the Crop Development Centre (CDC) at the University of Saskatchewan has developed cultivars such as CDC Amarillo (Warkentin et al., 2014a), CDC Limerick (Warkentin et al., 2014b), and CDC Inca (Warkentin et al., 2018) with improved SPC of up to 25%. It is well known in field peas that SPC is negatively correlated with grain yield (GY) (Tar’an et al., 2004). Although the SPC and yield have been improved in pea through different breeding strategies, the underlying molecular mechanisms controlling these complex traits are relatively unknown.

QTLs associated with SPC in pea will enable marker-assisted selection (MAS) to accelerate development of pea cultivars with improved protein content. A few studies have reported QTLs for SPC in pea. Krajewski et al. (2012) reported two QTLs with LOD values of 5.6 and 5.2 located on linkage group (LG) 5. Klein et al. (2020) used nine inter-connected pea RIL populations and identified 21 QTLs for SPC explaining the phenotypic variance from 4% to 22%. Meta-analysis of these QTLs identified six meta-QTLs for SPC in two to four environments. Gali et al. (2019) conducted a genome-wide association study (GWAS) and identified one locus on LG3 and two loci on LG5 associated with SPC. All of these studies indicated that SPC in pea is a complex quantitative trait. Most of these QTLs captured only small to moderate variability for SPC, and combined with variability across environments and negative correlation with yield, the SPC QTLs have not been used effectively in pea breeding programs. Seed protein QTLs have been identified in many crop species, including soybean, maize, wheat, and rice (Wang et al., 2021; Saini et al., 2022). In soybean, for example, QTLs associated with SPC have been mapped to specific chromosomes and were used to develop soybean varieties with higher SPC (Prenger et al., 2019). Similarly, in wheat, QTLs associated with gluten protein content have been identified (Li et al., 2023), which can be used to develop wheat varieties with improved bread-making properties.

Linkage analysis is a useful approach to dissect the genetic basis of complex traits in crop plants. With an objective of identifying and comparing the QTLs of SPC, diverse recombinant inbred line (RIL) populations were derived from crosses made between high protein and medium protein lines. Previously, we evaluated a bi-parental RIL population PR-25 under field trials in Saskatchewan, Canada from 2019 to 2021. PR-25 is a RIL population derived from the cross of two elite cultivars, CDC Amarillo and CDC Limerick. Three QTLs for SPC were reported from this population (Zhou et al., 2022). The genetic architecture of complex traits such as SPC is known to differ between the mapping populations based on their genetic background (Park et al., 2023). In the current study, we attempted to identify SPC QTLs in RIL populations PR-30 and PR-31 derived from different high SPC parents that differed significantly in their agronomic performance. The overall goal was to provide information that breeders can use for MAS to efficiently improve the nutritional quality of the pea crop.

## 2 Materials and methods

### 2.1 Mapping populations

Two diverse RIL populations, PR-30 (MP1918 × P0540-91) and PR-31 (Ballet × Cameor) arising from separate breeding programs, were used in the current study. PR-30 was developed at the Agriculture and Agri-Food Canada, Lacombe, Alberta, Canada. PR-31 is the “POP-4” population developed at the INRA, Dijon, France (Bourion et al., 2010; Bourgeois et al., 2011). Both populations were derived from crossing yellow cotyledon pea cultivars of moderate and high SPC. The high SPC breeding line P0540-91 and Cameor were used as pollen donors in the bi-parental crosses. A total of 166 RILs of PR-30 were used in the current study. A total of 176 RILs of PR-31 were used for phenotyping, out of which 159 RILs that were previously genotyped were used for QTL analysis (Tayeh et al., 2015).

### 2.2 Phenotyping

PR-30 and PR-31 populations were evaluated at two and three locations in 2020 and 2021, respectively. Rosthern and Lucky Lake in Saskatchewan were used as test locations for both years. Floral (Saskatoon) was the third location utilized in 2021. In each location, individual RILs were grown in 1-m^2^ microplots with three replications arranged in a randomized complete block design (RCBD).

Agronomic data including days to flower (DTF; 50% of the plants in a plot had fully opened flowers), plant height (PH; after complete flowering; cm), lodging (1–9 scale; in mid-pod development stage), and days to maturity (DTM; ~75% of the plants within the plot are matured) segregating in the population were recorded during the growing season. Seeds harvested from each plot were measured for total weight to calculate the GY of each plot was converted to kg/ha and used to measure the thousand seed weight (TSW) in grams. SPC and seed starch concentration (SSC) were measured using ~50 g of seeds harvested from each plot using a non-destructive method based on near-infrared (NIR) spectroscopy (Arganosa et al., 2006) using a FOSS NIR Systems 6500 NIR Spectrophotometer (Foss Tecator, Hoeganaes, Sweden). Analysis of variance (ANOVA) was conducted using PROC MIXED model in SAS 9.4 (SAS Institute Inc., NC, USA). Lines were considered as fixed effects while replications were considered as random effects. Locations were not the same in the 2 years of the field trials (Rosthern and Lucky Lake in 2020; Floral, Rosthern and Lucky Lake in 2021); therefore, location was substituted with station-year in the combined analysis. Correlation of SPC with other measured traits was calculated using the PROC.CORR in SAS. Broad sense heritability (*H* ^2^) was determined using ICImapping v 4.2 (Meng et al., 2015) on the basis of the mean across replications and environments.

### 2.3 Genotyping and development of linkage map

PR-30 was genotyped using the Axiom® 90K SNP Array developed by INRA, France and described by Ellis et al. (2023). The array was obtained from Thermo Fisher Scientific. Genotyping was conducted by Euroffins (WI, USA) using DNA extracted from young leaves of 10- to 14-day-old seedlings. The polymorphic SNP markers identified were filtered for segregation distortion (>90%) and missing values (>15%) and used for linkage map construction. The filtered polymorphic markers were binned using Icimapping v 4.2 (Meng et al., 2015; https://isbreedingen.caas.cn/software/qtllcimapping/294607.htm). The bin representative markers were used for linkage map construction by MstMap. The linkage groups were separated at a logarithm of odds ratio (LOD score; Morton, 1955) of 9.0. The map distance was calculated using the Kosambi function. The markers co-localized at each locus were filtered to select one SNP marker representing each unique locus for QTL analysis. The nomenclature of these markers represented the chromosome, linkage group, and base pair position of the corresponding SNP in the reference pea genome sequence of cv. Cameor (Kreplak et al., 2019). SNPs positioned on the non-chromosomal regions of Cameor genome were referred by their scaffold (Sc) and super scaffold (SSc) numbers.

The PR-31 population (POP-4) was earlier genotyped using a 13.2K SNP array, Genopea (Tayeh et al., 2015). We used the linkage map published by Tayeh et al. (2015) for QTL analysis in the current study. This linkage map is based on 6,797 polymorphic markers and represents 1,299 unique loci and covered a map distance of 861.8 cM in seven linkage groups (LG1 to LG7). The Axiom® 90K SNP array used for genotyping PR-30 includes the vast majority of the SNPs from Genopea; thus, many common markers were used for genotyping PR-30 and PR-31 populations. In the current study, the nomenclature of SNP markers on Genopea was modified to represent their chromosomal and base pair position in the reference pea genome of Cameor (Kreplak et al., 2019).

### 2.4 QTL analysis

The phenotypic means of PR-30 (**Table 1**) and PR-31 (**Table 2**) by location for SPC and GY were used for QTL mapping. The two parents of PR-31 population were also quite diverse for other traits including PH, DTM, TSW, and SSC compared to the parents of PR-30 population. These traits of PR-31 were also used for QTL mapping and to compare their co-localization with SPC and GY QTLs. QTL mapping was performed using composite interval mapping (CIM) using QTL Cartographer v2.5 (Wang et al., 2007). The QTL search was performed along the linkage groups using standard model 6 based on both forward and backward regression and a walk distance of 2.0 cM. To declare a QTL, the threshold for each search was obtained from 1,000 permutations with a significance level of 0.05. The QTL analysis was performed using the SPC and GY data from each station-year, as well the combined data from all five station-years.

**Table 1:** Summary of the individual station-year statistical analysis of selected traits of RIL population, PR-30 (MP1918 × P0540-91; 166 lines) evaluated under field conditions in five station-years with three replicates per location.

Station-year/Trait	MP1918	P0540-91	RILs					
	Mean	Mean	*F*-value	Min	Max	Mean	SD	CV
2020 Rosthern								
Days to flower	54	53	8.5***	47	60	54	1.9	3.5
Plant height (cm)	110	92	1.6***	58	128	98	11.5	11.7
Days to maturity	88	87	1.6***	81	96	88	3.3	3.7
Grain yield (kg/ha)	5,516	3,520	1.3*	2,210	7,282	4,507	1,010.3	22.4
Thousand seed weight (g)	218	213	22.9***	182	291	238	19.3	8.1
Seed starch conc. (%)	51.5	38.3	19.4***	33.9	55.9	49.2	3.7	7.6
2020 Lucky Lake								
Days to flower	56	54	9.6***	48	61	55	1.8	3.3
Plant height (cm)	96	88	1.1ns	57	122	95	41.4	43.6
Days to maturity	90	89	2.6***	84	98	90	2.3	2.5
Grain yield (kg/ha)	3,300	1,915	2.0***	1,172	5,813	3,272	831.0	25.4
Thousand seed weight (g)	217	232	6.0***	182	287	239	21.0	8.8
Seed starch conc. (%)	58.6	46.8	4.6***	36.6	66.1	57.9	5.3	9.2
2021 Floral								
Days to flower	55	53	7.6***	50	58	55	1.5	2.8
Plant height (cm)	57	52	4.2***	41	74	56	6.0	10.8
Days to maturity	91	92	3.0***	86	95	91	2.1	2.3
Grain yield (kg/ha)	3,263	2,233	2.9***	1,491	4,510	2,902	492.5	17.0
Thousand seed weight (g)	218	236	5.7***	200	386	246	21	8.4
Seed starch conc. (%)	52.2	41.0	11.8***	35.0	55.4	49.3	3.2	6.5
2021 Rosthern								
Days to flower	50	50	4.3***	46	52	49	1.4	2.8
Plant height (cm)	46	44	2.6***	29	78	49	8.7	17.8
Days to maturity	77	80	4.0***	73	84	79	2.4	3.0
Grain yield (kg/ha)	1,297	861	1.4**	223	3,723	1,423	708.3	49.8
Thousand seed weight (g)	186	223	9.4***	181	287	227	19.6	8.7
Seed starch conc. (%)	49.8	37.9	9.6***	34.3	57.2	49.7	4.0	8.0
2021 Lucky Lake								
Days to flower	50	50	2.0***	48	56	51	1.9	3.7
Plant height (cm)	44	43	6.4***	32	62	47	5.8	12.4
Days to maturity	78	79	6.4***	75	86	80	2.1	2.6
Grain yield (kg/ha)	1,757	1,133	2.0***	500	2,281	1,419	328.6	23.2
Thousand seed weight (g)	210	230	12.2***	191	295	241	19.7	8.2
Seed starch conc. (%)	50.4	40.7	15.5***	34.9	54.9	49.4	3.3	6.6

ns, not significant; *p < 0.05; **p < 0.01; ***p < 0.001; SD, standard deviation; CV, coefficient of variation (%).

**Table 2:** Summary of the individual station-year statistical analysis of selected traits of RIL population, PR-31 (Ballet × Cameor; 176 lines) evaluated under field conditions in five station-years with three replicates per location.

Station-year/Trait	Ballet	Cameor	RILs					
	Mean	Mean	*F*-value	Min	Max	Mean	SD	CV
2020 Rosthern								
Days to flower	49	44	13.4***	40	53	47	2.3	4.9
Plant height (cm)	76	48	4.3***	27	103	61	12.1	20.0
Days to maturity	87	79	2.9***	75	93	82	2.7	3.3
Grain yield (kg/ha)	3,885	2,551	2.7***	768	4,950	2,910	704.9	24.2
Thousand seed weight (g)	225	193	25.1***	164	291	214	20.4	9.6
Seed starch conc. (%)	49.0	46.3	5.5***	41.2	52.7	47.9	2.0	4.1
2020 Lucky Lake								
Days to flower	48	50	8.4***	43	57	49	2.5	5.1
Plant height (cm)	54	66	4.1***	27	94	57	10.5	18.5
Days to maturity	85	89	2.8***	78	98	87	3.3	3.8
Grain yield (kg/ha)	2,148	2,085	1.6***	759	4,043	2,089	631.1	30.2
Thousand seed weight (g)	198	236	19.4***	157	286	221	20.4	9.2
Seed starch conc. (%)	48.7	45.4	5.7***	40.2	54.8	47.9	2.3	4.7
2021 Floral								
Days to flower	51	45	17.6***	44	54	49	2.7	5.5
Plant height (cm)	42	27	4.4***	15	60	36	6.0	16.7
Days to maturity	88	76	2.8***	75	94	86	5.1	5.9
Grain yield (kg/ha)	1,570	817	6.1***	356	3,127	1,440	490.0	34.0
Thousand seed weight (g)	242	193	9.5***	182	283	231	19.0	8.2
Seed starch conc. (%)	46.2	44.8	5.9***	39.3	52.5	46.1	2.1	4.6
2021 Rosthern								
Days to flower	48	45	7.3***	41	53	46	2.2	4.7
Plant height (cm)	42	37	2.3***	19.0	66.0	34.5	6.9	20.0
Days to maturity	84	77	2.8***	72	90	82	2.4	3.0
Grain yield (kg/ha)	974	1,116	1.9***	94	3,819	803	497.5	61.9
Thousand seed weight (g)	237	182	6.3***	164	272	220	21.2	9.6
Seed starch conc. (%)	46.0	45.9	3.9***	37.6	55.1	46.6	2.6	5.5
2021 Lucky Lake								
Days to flower	50	44	7.9***	41	53	47	2.7	5.8
Plant height (cm)	32	27	3.3***	14	54	31	6.0	19.1
Days to maturity	86	76	3.1***	72	89	82	3.6	4.4
Grain yield (kg/ha)	905	285	4.1***	97	1,466	744	259.0	34.8
Thousand seed weight (g)	246	185	11.4***	158	277	223	20.5	9.2
Seed starch conc. (%)	48.6	47.1	6.4***	42.5	53.9	48.6	2.2	4.5

***p < 0.001; SD, standard deviation; CV, coefficient of variation (%).

## 3 Results

### 3.1 Phenotyping for SPC

Analysis of variance (ANOVA) in combined (five station-years) data analysis showed significant differences (*p* < 0.001) for SPC among the lines of PR-30 and PR-31 populations (**Tables 3** and **4**). The effects of station-year, as well as the line × station-year interaction, were significant (*p* < 0.001) for the RIL populations. Thus, data were presented separately for each station-year.

**Table 3:** *F*-values and summary of the statistical analysis from the analysis of variance for traits of RIL population, PR-30 (MP1918 × P0540-91; 166 lines), evaluated under field conditions in five station-years with three replicates per location.

Trait	*F*-value			Min	Max	Mean	SD	CV	*H* ^2^
	Line	Station-year	Line × station-year						
Seed protein conc. (%)	6.5***	563.4***	1.3***	20.7	30.1	25.3	1.6	6.2	0.82
Days to flower	19.5***	2,509.1***	1.4***	46.0	61.0	52.8	2.8	5.4	0.93
Plant height (cm)	6.3***	5,792.7***	1.1ns	29.0	128.0	68.6	24.0	35.0	0.83
Days to maturity	8.2***	4,254.8***	1.3***	73.0	98.0	85.6	5.6	6.6	0.84
Grain yield (kg/ha)	3.2***	1,981.7***	1.1*	223	7282	2702	1377	51.0	0.66
Thousand seed weight (g)	48.0***	249.5***	2.2***	180.9	386.2	238.3	20.3	8.5	0.96
Seed starch conc. (%)	29.1***	1,349.8***	1.1*	33.9	66.1	51.1	5.2	10.2	0.96

Five station-years (2020 Rosthern; 2020 Lucky Lake; 2021 Floral; 2021 Rosthern; 2021 Lucky Lake); ns, not significant; *p < 0.05; ***p < 0.001; SD, standard deviation; CV, coefficient of variation (%), H^2^ Broad sense heritability on the basis of the mean across replications and environments. The minimum and maximum values of each trait presented are the observed values compared between all the RILs and their replications.

**Table 4:** *F*-values and summary of the statistical analysis from the analysis of variance for traits of RIL population, PR-31 (Ballet × Cameor; 176 lines), evaluated under field conditions in five station-years with three replicates per location.

Trait	*F*-value			Min	Max	Mean	SD	CV	*H* ^2^
	Line	Station-year	Line × station-year						
Seed protein conc. (%)	20.4***	1,753.1***	2.3***	20.6	33.2	27.4	2.2	7.9	0.89
Days to flower	39.5***	705.7***	2.1***	40.0	57.0	47.4	2.8	6.0	0.94
Plant height (cm)	13.2***	2,657.4***	1.6***	14.0	103.0	41.8	14.4	34.3	0.87
Days to maturity	6.1***	345.8***	2.0***	72.0	98.0	83.6	4.1	5.0	0.72
Grain yield (kg/ha)	6.3***	2,242.8***	1.6***	94	4847	1465	937	63.9	0.77
Thousand seed weight (g)	52.1***	218.3***	3.0***	158.0	291.0	221.9	21.1	9.5	0.95
Seed starch conc. (%)	19.1***	268.6***	1.7***	37.6	55.1	47.4	2.5	5.2	0.91

Five station-years (2020 Rosthern; 2020 Lucky Lake; 2021 Floral; 2021 Rosthern; 2021 Lucky Lake); ns, not significant; ***p < 0.001; SD, standard deviation; CV, coefficient of variation (%); H^2^, Broad sense heritability on the basis of the mean across replications and environments. The minimum and maximum values of each trait presented are the observed values compared between all the RILs and their replications.

**Figure 1:**
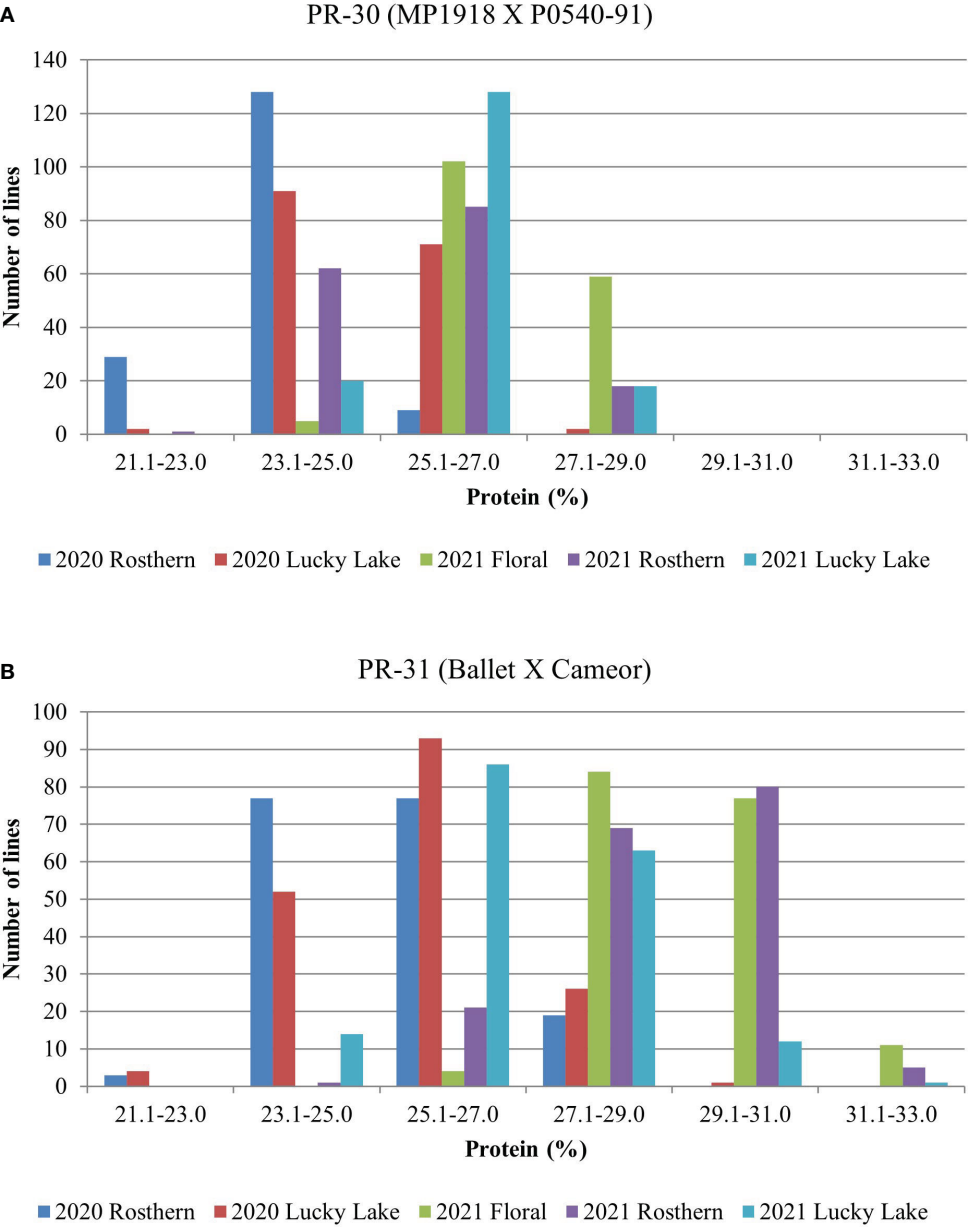
Frequency distribution of **(A)** PR-30 (MP1918 × P0540-91; 166 lines) and **(B)** PR-31 (Ballet × Cameor; 176 lines) RIL populations for seed protein concentration measured in five station-years with three replicates per location.

Station-year wise, the effect of the line was significant for both RIL populations at Floral, Rosthern, and Lucky Lake locations (**Table 5**). For PR-30 population, the SPC varied from 20.7% (2020 Lucky Lake) to 30.1% (2021 Floral) (**Table 5**; **Figure 1**). The SPCs for the two parents of PR-30, MP1918 and P0540-91 were 24.7% and 26.9%, respectively. For PR-31 population, the SPC ranged from 20.6% (2020 Lucky Lake) to 33.2% (2021 Rosthern). The mean SPCs of Ballet and Cameor were 25.8% and 27.9%, respectively.

**Table 5:** Summary of the individual station-year statistical analysis of seed protein concentration of RIL populations, PR-30 (MP1918 × P0540-91) and PR-31 (Ballet × Cameor), evaluated under field conditions in five station-years with three replicates per location.

Population	Station-year	Parent/RILs	Mean SPC	*F*-value	Min	Max	SD	CV
PR-30	2020 Rosthern	MP1918	22.7		22.0	23.4	0.5	
		P0540-91	24.8		23.9	26.5	0.9	
		RILs	23.7	2.84***	20.9	26.7	1.0	4.1
	2020 Lucky Lake	MP1918	24.5		23.8	26.1	0.9	
		P0540-91	26.1		25.3	27.0	0.7	
		RILs	25.0	2.46***	20.7	28.0	1.1	4.5
	2021 Floral	MP1918	25.0		23.3	26.2	1.0	
		P0540-91	28.4		27.3	29.3	0.7	
		RILs	26.7	2.9***	23.4	30.1	1.2	4.5
	2021 Rosthern	MP1918	26.0		25.0	27.6	0.9	
		P0540-91	28.0		26.7	30.4	1.3	
		RILs	25.5	2.1***	21.2	30.0	1.8	6.9
	2021 Lucky Lake	MP1918	25.6		24.7	26.9	0.8	
		P0540-91	27.0		25.6	27.9	0.9	
		RILs	25.9	4.9***	23.3	29.4	0.9	3.7
PR-31	2020 Rosthern	Ballet	23.7		22.7	26.2	1.3	
		Cameor	26.1		22.8	27.4	1.7	
		RILs	25.3	7.72***	21.5	29.9	1.4	5.5
	2020 Lucky Lake	Ballet	23.8		22.5	25.2	1.0	
		Cameor	27.3		25.6	28.9	1.1	
		RILs	25.7	5.07***	20.6	30.4	1.6	6.3
	2021 Floral	Ballet	27.8		27.0	28.6	0.6	
		Cameor	29.6		28.5	30.3	0.6	
		RILs	29.1	8.3***	25.3	32.7	1.3	4.6
	2021 Rosthern	Ballet	27.5		25.9	29.0	1.3	
		Cameor	29.1		27.8	30.5	0.9	
		RILs	28.8	3.9***	23.3	33.2	1.8	6.2
	2021 Lucky Lake	Ballet	26.0		25.3	26.8	0.7	
		Cameor	27.6		26.7	28.5	0.7	
		RILs	26.9	9.4***	23.0	31.7	1.5	5.7

***p < 0.001; SPC, seed protein concentration; SD, standard deviation; CV, coefficient of variation (%).

### 3.2 Phenotyping for agronomic and yield traits

For agronomic traits (DTF, PH, and DTM), GY, TSW, the effects of line, station-year, and line × station-year were significant (*p* < 0.05) for all of the traits of PR-30 and PR-31 except for PH in PR-30 (**Tables 3** and **4**).

Similarly, station-year wise, the effect of line was significant for most of the evaluated traits (**Tables 1** and **2**). For these populations, a wide range of variation was observed for agronomic traits, GY, TSW, and SSC (**Tables 1** and **2**).

### 3.3 Correlation of SPC with other traits

Pearson correlation analysis indicated significant (<0.05) positive correlation of SPC with DTF and DTM, whereas correlation of SPC was negative with GY and SSC for PR-30 (**Table 6**). Like PR-30, SPC was negatively correlated with GY and SSC for PR-31 (**Table 7**).

**Table 6:** Pearson correlation coefficients for traits of RIL population, PR-30 (MP1918 × P0540-91; 166 lines) evaluated under field conditions in five station-years with three replicates per location.

Trait	SPC	DTF	PH	DTM	GY	TSW	SSC
SPC	1.0						
DTF	0.25**	1.0					
PH	0.12ns	0.66***	1.0				
DTM	0.41***	0.74***	0.62***	1.0			
GY	−0.21**	0.30***	0.52***	0.20*	1.0		
TSW	−0.05ns	0.18*	0.28***	0.23**	0.31***	1.0	
SSC	−0.65***	0.07ns	0.16*	−0.1ns	0.40***	0.37***	1.0

Five station-years (2020 Rosthern; 2020 Lucky Lake; 2021 Floral; 2021 Rosthern; 2021 Lucky Lake); ns, not significant; *p < 0.05; **p < 0.01; ***p < 0.001.

SPC, Seed protein concentration (%); DTF, Days to flowering; PH, Plant height (cm); DTM, Days to maturity; GY, Grain yield (kg/ha); TSW, Thousand seed weight (g); SSC, Seed starch concentration (%).

**Table 7:** Pearson correlation coefficients for traits of RIL population, PR-31 (Ballet × Cameor; 176 lines) evaluated under field conditions in five station-years with three replicates per location.

	SPC	DTF	PH	DTM	GY	TSW	SSC
SPC	1.0						
DTF	−0.46***	1.0					
PH	−0.26***	0.24**	1.0				
DTM	0.04ns	0.49***	0.13ns	1.0			
GY	−0.66***	0.54***	0.39***	0.01ns	1.0		
TSW	0.24**	0.06ns	−0.20ns	0.36***	−0.07ns	1.0	
SSC	−0.74***	0.44***	0.32***	0.01ns	0.56***	−0.12ns	1.0

Five station-years (2020 Rosthern; 2020 Lucky Lake; 2021 Floral; 2021 Rosthern; 2021 Lucky Lake); ns, not significant; **p < 0.01; ***p < 0.001.

SPC, Seed protein concentration (%); DTF, Days to flowering; PH, Plant height (cm); DTM, Days to maturity; GY, Grain yield (kg/ha); TSW, Thousand seed weight; SSC, Seed starch concentration (%).

### 3.4 Genotyping and development of linkage map

**Figure 2:**
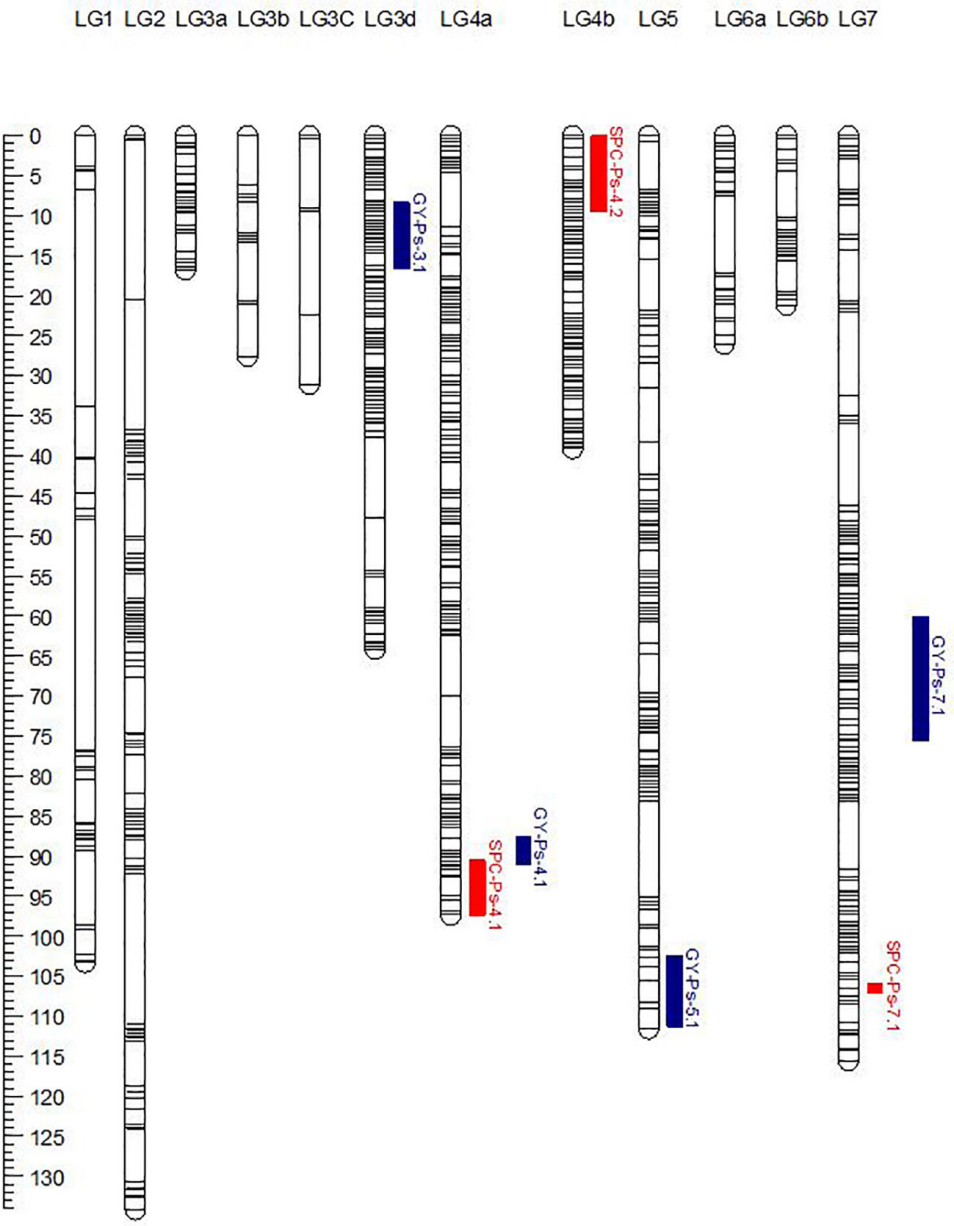
Genetic linkage map of the PR-30 (MP1918 × P0540-91) RIL population. The genetic positions of QTLs for seed protein concentration (SPC) and grain yield (GY) were represented on the linkage map.

PR-30 population was genotyped using an Axiom® 90K SNP array that resulted in the identification of 14,986 polymorphic SNP markers after filtering for segregation distortion and missing values. These SNP markers were binned using ICimapping and were grouped to 4,835 bins. The bin representative markers were used for linkage mapping using Mstmap. At an LOD value of 9.0, these markers were grouped into 12 linkage groups (LG1, LG2, LG3a, LG3b, LG3c, LG3d, LG4a, LG4b, LG5, LG6a, LG6b, and LG7) to represent 708 unique loci and a map distance of 788.0 cM (**Table 8**; **Figure 2**). The published linkage map of PR-31 (Tayeh et al., 2015) was used for QTL analysis in this study. In both mapping populations, the grouping of the SNP markers into linkage groups and the order of markers within the linkage groups were comparable with the physical position of these markers in the pea genome sequence (Kreplak et al., 2019). The order of markers in PR-30 and PR-31 linkage maps is provided in **Supplementary File 1**.

**Table 8:** Details of genetic linkage map of PR-30 RIL population (MP1918 × P0540-91).

Chromosome/Linkage group	No. of unique loci mapped	Map distance (cM)	Average marker distance (cM)	Standard deviation	Max. distance between markers (cM)
Chr 2/LG1	32	103.28	3.3	6.9	28.8
Chr 6/LG2	82	134.15	1.7	3.6	19.8
Chr 5/LG3a	26	16.8	0.7	0.5	2.4
Chr 5/LG3b	12	27.64	2.5	2.9	7.4
Chr 5/LG3c	6	31.11	6.2	5.6	12.9
Chr 5/LG3d	88	64.17	0.7	1.3	10.1
Chr 4/LG4a	121	97.26	0.8	1.1	7.3
Chr 4/LG4b	71	39.11	0.6	0.3	1.7
Chr 3/LG5	100	111.63	1.1	1.7	11.9
Chr 1/LG6a	23	26.14	1.2	1.9	9.6
Chr 1/LG6b	23	21.16	1.0	1.3	5.9
Chr 7/LG7	124	115.55	1.0	1.7	10.5

### 3.5 QTL identification

The genetic linkage map of PR-30 summarized in **Table 8** in combination with the SPC of PR-30 RILs measured in five station-years in 2020 and 2021 was used for identification of SPC and GY-related QTLs. Based on the least square mean of SPC in five replicated trials, three QTLs named *SPC-Ps-4.1*, *SPC-Ps-4.2*, and *SPC-Ps-7.1* were identified in PR-30 (**Table 9**; **Figure 2**). *SPC-Ps-4.1* located on LG4a (chromosome 4) has an LOD score of 5.7 and explained 12.1% of the phenotypic variance. *SPC-Ps-4.2* located on LG4b (chromosome 4) has an LOD score of 7.2 and explained 14.5% of the phenotypic variance. These two QTLs have negative additive effects of −0.24 and −0.26, respectively, indicating that they were inherited from the high protein parent P0540-91 used as pollen donor in developing this mapping population. The third QTL *SPC-Ps-7.1* is located on LG7 (chromosome 7). This QTL has an LOD score of 2.8 and explained a phenotypic variance of 6.6%. This QTL was inherited from the moderate SPC parent MP1918. When compared between individual station-years, *SPC-Ps-4.1* was significant in one station-year, while *SPC-Ps-4.2* and *SPC-Ps-7.1* were significant in three of the five station-years (**Table 9**).

**Table 9:** QTLs for seed protein concentration and grain yield detected in pea RIL population PR-30 (MP1918 × P0540-91) evaluated in five station-years in Saskatchewan, Canada (2020–2021).

Trait	QTL	Chromosome LG	QTL interval/peak position (cM)	QTL flanking markers	LOD score	*R* ^2^ (%)	Additive effect*
SPC	*SPC-Ps-4.1* ^a^	Chr4/LG4a	90.5–97.4/97.0	Chr4LG4_233423219–Chr4LG4_287277057	5.67	12.1	-0.24
	*SPC-Ps-4.2* ^b^	Chr4/LG4b	0–9.5/1.2	Chr4LG4_285140945–Chr4LG4_305099926	7.22	14.5	−0.26
	*SPC-Ps-7.1* ^c^	Chr7/LG7	105.9–107.1/106.5	Chr7LG7_433290731–Chr7LG7_441692688	2.8	6.6	0.18
GY	*GY-Ps-3.1* ^d^	Chr5/LG3d	8.3–16.7/10.7	Chr5LG3_303358259–Chr5LG3_462246878	4.65	11.04	107.27
	*GY-Ps-4.1* ^e^	Chr4/LG4a	87.5–91.1/88.7	Chr4LG4_206705452–Chr4LG4_244379998	3.44	7.27	−90.2
	*GY-Ps-5.1* ^f^	Chr3/LG5	102.4–111.3/106.5	Chr3LG5_455814220–Chr3LG5_436109557	3.59	8.5	−99.96
	*GY-Ps-7.1* ^g^	Chr7/LG7	60.1–75.6/63.7	Chr7LG7_120343438–Chr7LG7_165855078	6.07	12.48	118.34

LG, linkage group; The QTL identification is based on the phenotypes measured in five station-years—Floral 2021, Lucky Lake 2020 and 2021, and Rosthern 2020 and 2021. *A negative additive effect indicates that the QTL is introgressed from the high protein parent P0540-91 and a positive additive effect indicates the introgression of QTL from the moderate protein parent MP1918.

For seed protein concentration (SPC) and grain yield (GY) QTLs, the QTL effect significant in individual station-years is also indicated: ^a^Floral 2021, ^b^Lucky Lake 2020, Lucky Lake 2021 and Rosthern 2021; ^c^Floral 2021, Rosthern 2021 and Lucky Lake 2021, ^d^Floral 2021 and Rosthern 2021, ^e^Rosthern 2020, ^f^Floral 2021, and ^g^Floral 2021 and Rosthern 2020.

Four significant QTLs were identified to be associated with GY in PR-30. These QTLs named *GY-Ps-3.1*, *GY-Ps-4.1*, *GY-Ps-5.1*, and *GY-Ps-7.1* were located on linkage groups 3d, 4a, 5 and 7, respectively (**Table 9**). These QTLs had an LOD score of 3.4 to 6.1 and explained a phenotypic variation of 7.3% to 12.5%. *GY-Ps-3.1* and *GY-Ps-7.1* were derived from the moderate SPC parent MP1918 and explained a phenotypic variation of 11.0% and 12.5%, respectively. The QTL *GY-Ps-4.1* has a partial overlap with *SPC-Ps-4.1* (**Table 9**; **Figure 2**). Though these two QTLs on LG4a were derived from P0540-91, the peak regions of these QTLs were separated by 8.3 cM (**Table 9**).

**Figure 3:**
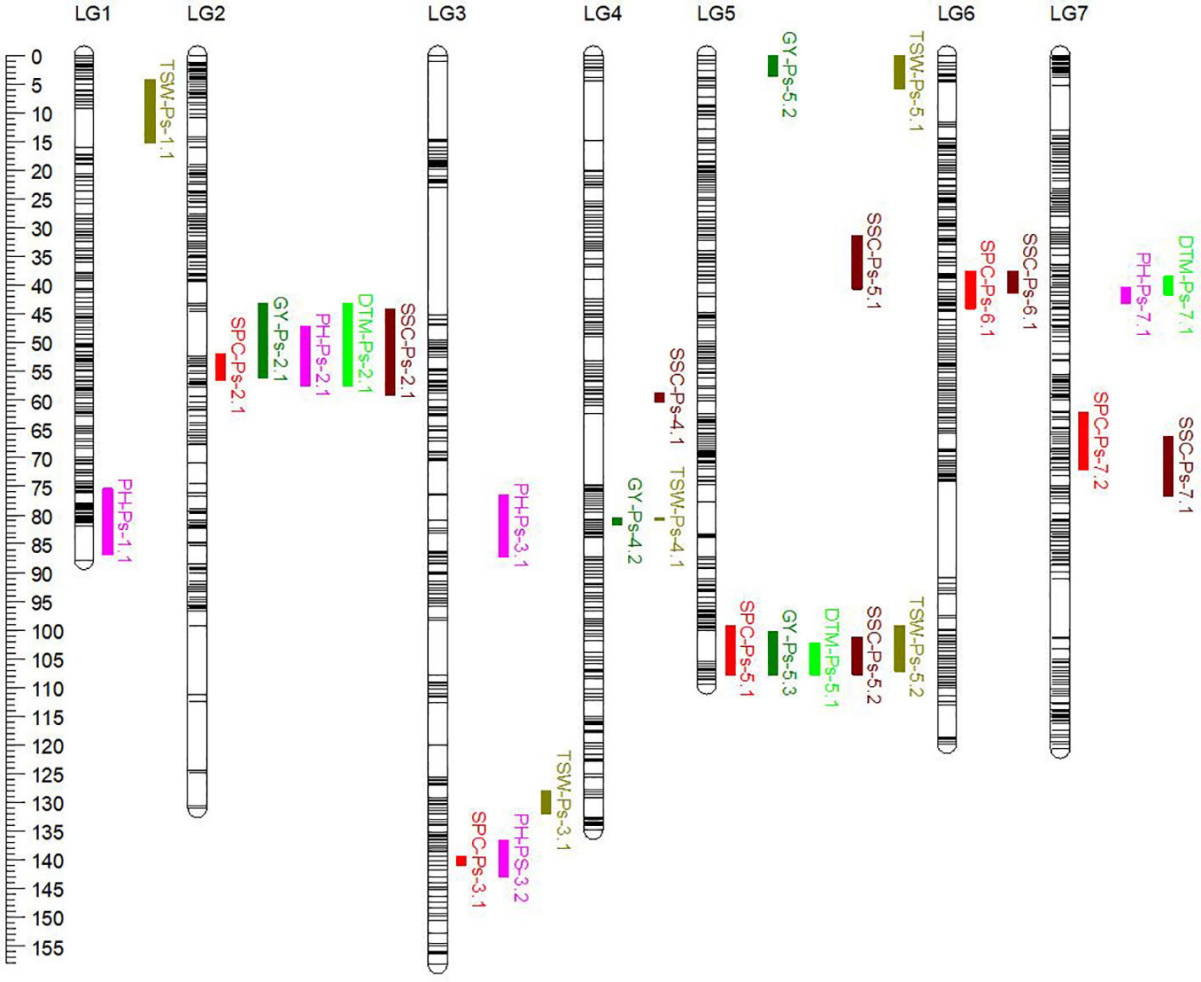
Genetic linkage map of the PR-31 (Ballet × Cameor) RIL population. The genetic positions of QTLs for seed protein concentration (SPC), grain yield (GY), plant height (PH), days to maturity (DTM), thousand seed weight (TSW), and seed starch concentration (SSC) were represented on the linkage map in different colors.

The genetic linkage map of PR-31, representing 1,299 unique loci in combination with the SPC and GY of PR-31 RILs measured in five station-years in 2020 and 2021, was used for QTL analysis. Based on the least square mean of SPC measured in five replicated trials, five QTLs associated with SPC were identified in PR-31 (**Table 10**; **Figure 3**). These QTLs located on linkage groups 2, 3, 5, 6, and 7 were named *SPC-Ps-2.1*, *SPC-Ps-3.1*, *SPC-Ps-5.1*, *SPC-Ps-6.1*, and *SPC-Ps-7.2*, respectively. *SPC-Ps-7.2* has the highest LOD score of 11.3 and explained 17.2% of the phenotypic variance, followed by *SPC-Ps-5.1*, which has an LOD score of 7.9 and explained 11.6% of the phenotypic variance. Both these QTLs were also significant in three and four of the five station-years tested, respectively. Based on the additive effect of QTLs, *SPC-Ps-5.1* was derived from Ballet, and the other four QTLs including *SPC-Ps-7.2* were derived from Cameor.

**Table 10:** QTLs for multiple traits measured in pea RIL line population PR-31 (Ballet × Cameor) evaluated in five station-years in Saskatchewan, Canada (2020–2021).

Trait	QTL	Chromosome/LG	QTL interval/Peak position (cM)	QTL flanking markers	LOD score	*R* ^2^ (%)	Additive effect*
SPC	*SPC-Ps-2.1* ^a^	Chr6/LG2	51.9–56.5/55.5	Chr6LG2_109241711–Chr6LG2_166113140	4.7	6.6	−0.30
	*SPC-Ps-3.1*	Chr5/LG3	139.4–140.9/140.9	Chr5LG3_526923067–Chr5LG3_535182050	3.5	4.9	−0.25
	*SPC-Ps-5.1* ^b^	Chr3/LG5	99.2–107.8/106.0	Chr3LG5_404463049–PsCam029064_17292_723/Chr3LG5_436484518	7.9	11.6	0.40
	*SPC-Ps-6.1*	Chr1/LG6	37.6–44.1/39.3	Chr1LG6_85954546–Chr1LG6_122850728	4.2	5.9	−0.28
	*SPC-Ps-7.2* ^c^	Chr7/LG7	62.1–72.2/65.5	Chr7LG7_310787199–Chr7LG7_338413048	11.3	17.2	−0.48
GY	*GY-Ps-2.1* ^d^	Chr6/LG2	43.2–56.1/52.9	Chr6LG2_65909284–PsCam012060_8218_982	8.2	15.4	125.62
	*GY-Ps-4.2*	Chr4/LG4	80.5–81.7/81.3	Chr4LG4_202897225–Chr4LG4_208528599	3.1	5.0	−71.01
	*GY-Ps-5.2* ^e^	Chr3/LG5	0.0–3.7/3.0	Chr3LG5_854384–Chr3LG5_7077759	3.6	6.4	78.48
	*GY-Ps-5.3* ^f^	Chr3/LG5	100.2–107.8/106.0	Chr3LG5_413900293–PsCam029064_17292_723/Chr3LG5_436484518	5.0	9.2	−96.09
PH	*PH-Ps-1.1*	Chr2/LG1	75.4–86.9/81.0	Chr2LG1_400580254–Chr2LG1_409462314	12.2	17.4	2.4
	*PH-Ps-2.1*	Chr6/LG2	47.2–57.5/52.8	Chr6LG2_109241711–Chr6LG2_169432798	8.1	10.7	1.96
	*PH-Ps-3.1*	Chr5/LG3	76.5–87.4/82.7	Chr5LG3_201898831–Chr5LG3_238420050	11.3	15.8	2.33
	*PH-PS-3.2*	Chr5/LG3	136.5–143.0/138.6	Chr5LG3_512802583–AB53/Chr5LG3_547677746	4.6	5.9	1.41
	*PH-Ps-7.1*	Chr7/LG7	40.4–43.2/41.2	AD56/Chr7LG7_138688630–Chr7LG7_154133754	3.4	4.2	−1.20
DTM	*DTM-Ps-2.1*	Chr1/LG2	43.2–57.5/52.3	Chr6LG2_65909284–Chr6LG2_169432798	8.7	18.5	0.78
	*DTM-Ps-5.1*	Chr3/LG5	102.2–107.8/105.8	Chr3LG5_413900293–PsCam029064_17292_723/Chr3LG5_436484518	3.4	6.8	0.46
	*DTM-Ps-7.1*	Chr7/LG7	38.5–41.7/41.2	Chr7LG7_128033255–Chr7LG7_151329594	3.2	6.0	0.43
SSC	*SSC-Ps-2.1*	Chr6/LG2	44.2–59.1/55.5	Chr6LG2_69971193–Chr6LG2_171896410	11.3	19.4	0.75
	*SSC-Ps-4.1*	Chr4/LG4	58.8–60.4/59.0	Chr4LG4_132478004–Chr4LG4_137652225	3.7	5.8	0.40
	*SSC-Ps-5.1*	Chr3/LG5	31.4–40.7/36.8	PsCam001090_925_903/Chr3LG5_75172039–Chr3LG5_110292500	4.6	7.0	−0.44
	*SSC-Ps-5.2*	Chr3/LG5	101.2–107.8/106.7	Chr3LG5_413900293–PsCam029064_17292_723/Chr3LG5_436484518	3.7	5.1	−0.39
	*SSC-Ps-6.1*	Chr1/LG6	37.6–41.5/38.2	Chr1LG6_85954546–Chr1LG6_97387872	4.5	7.0	0.44
	*SSC-Ps-7.1*	Chr7/LG7	66.4–76.7/71.7	AA317/Chr7LG7_320407028–Chr7LG7_358940625	8.5	14.3	0.63
TSW	*TSW-Ps-1.1*	Chr2/LG1	4.2–15.3/10.3	Chr2LG1_9082046–Chr2LG1_24785378	6.5	11.8	6.20
	*TSW-Ps-3.1*	Chr5/LG3	128.0–131.9/130.4	Chr5LG3_484210886–Chr5LG3_502622225	4.8	8.3	5.08
	*TSW-Ps-4.1*	Chr4/LG4	80.5–80.9/80.8	Chr4LG4_202897225–Chr4LG4_203959915	3.0	4.4	3.81
	*TSW-Ps-5.1*	Chr3/LG5	0.0–5.9/0.0	Chr3LG5_854384–Sc01560_24800	4.5	7.5	4.85
	*TSW-Ps-5.2*	Chr3/LG5	99.2–107.2/102.2	Chr3LG5_404463049–Chr3LG5_434445642	8.4	18.1	7.54

LG, linkage group; SPC, Seed protein concentration (%); GY, Grain yield (kg/ha); PH, Plant height (cm); DTM, Days to maturity; SSC, Seed starch concentration (%); TSW, Thousand seed weight (g); The QTL identification is based on the phenotypes measured in five station-years—Floral 2021, Lucky Lake 2020 and 2021, and Rosthern 2020 and 2021. *A negative additive effect indicates that the QTL is introgressed from the high protein parent Cameor and a positive additive effect indicates the introgression of QTL from the moderate protein parent Ballet.

For SPC and GY QTLs, the QTL effect significant in individual station-years is also indicated: ^a^Floral 2021 and Lucky Lake 2021; ^b^Rosthern 2020, Floral 2021, Lucky Lake 2021 and Rosthern 2021; ^c^Rosthern 2020, Lucky Lake 2021 and Rosthern 2021; ^d^Rosthern 2020, Floral 2021 and Lucky Lake 2021; ^e^Floral 2021; ^f^Rosthern 2020, Floral 2021 and Rosthern 2021.

Four QTLs associated with GY, *GY-Ps-2.1*, *GY-Ps-4.2*, *GY-Ps-5.2*, and *GY-Ps-5.3*, were identified (**Table 10**). *GY-Ps-2.1* located on LG2 had an LOD score of 8.2 and explained 15.4% of the phenotypic variation. This QTL and *GY-Ps-5.2* were contributed by Ballet. *GY-Ps-4.1* identified in PR-30 and *GY-Ps-4.2* identified in PR-31 have partially overlapping positions on LG4, and their peak regions were identical, as determined by comparing the position of flanking markers on the pea reference genome sequence. The QTL interval of *GY-Ps-2.1* on LG2 (43.2-56.1 cM) overlapped with *SPC-Ps-2.1* (51.9-56.5 cM) in the PR-31 population; however, the additive effect of these QTLs differed in that *GY-Ps-2.1* is contributed by Ballet and *SPC-PS-2.1* is contributed by Cameor. A similar phenomenon was observed by comparing the QTLs *GY-Ps-5.3* and *SPC-Ps-5.1*. The QTL *GY-Ps-5.3* explained 9.2% of the phenotypic variance and was contributed by Cameor. This QTL overlapped with SPC-Ps-5.1 contributed by Ballet and the peak regions of both these QTLs are the same (**Table 10**). The co-localization of both these sets of protein and yield QTLs, with contrasting effect on protein and yield depending on inheritance of these QTLs from either of the parents, further supports the general trend of poor correlation between SPC and GY.

Five QTLs associated with PH were identified in the PR-31 population. These QTLs were located on linkage groups 1, 2, 3, and 7, with LOD scores ranging from 3.4 to 12.2 (**Table 10**). QTLs *PH-Ps-2.1* and *PH-Ps-3.2* with LOD scores of 8.1 and 4.6 co-localized with *SPC-Ps-2.1* and *SPC-Ps-3.1*, respectively. However, the additive effect of these PH QTLs was the opposite of the additive effect of corresponding SPC QTLs, indicating that the origin of these QTLs from Ballet increased the PH and reduced the SPC. The QTL *PH-Ps-2.1* also co-localized with *GY-Ps-2.1*.

Three QTLs associated with DTM were identified in the PR-31 population (**Table 10**). *DTM-Ps-2.1* with an LOD score of 8.7 co-localized with *SPC-Ps-2.1* and *GY-Ps-2.1*, while *DTM-Ps-5.1* co-localized with *SPC-Ps-5.1* and *GY-Ps-5.3*. A change of the additive effect of these co-localized QTLs from a positive to a negative value or vice versa depending on the trait was observed. For example, introgression of *SPC-Ps-2.1* QTL region from Ballet had a negative effect on SPC and a positive effect on DTM and yield to enhance these traits. Introgression of *SPC-Ps-5.1* from Ballet increased the DTM and SPC, but negatively affected the yield. Five QTLs associated with TSW, with LOD scores of 3.0 to 8.4, were identified in PR-31 (**Table 10**). QTL *TSW-Ps-4.1* co-localized with *GY-Ps-4.2* with a contrasting additive effect reflecting the negative correlation between TSW and GY. In contrast, *TSW-PS-5.1* and *GY-Ps-5.2* co-localized with a synergistic additive effect. *TSW-Ps-5.2* co-localized with both *SPC-Ps-5.1* and *GY-Ps-5.3* with varying additive effects.

Six QTLs associated with SSC were identified on linkage groups 2, 4, 5, 6, and 7 of the PR-31 population (**Table 10**). Four of these six QTLs, *SSC-Ps-2.1*, *SSC-PS-5.2*, *SSC-Ps-6.1*, and *SSC-Ps-7.1*, co-localized with *SPC-Ps-2.1*, *SPC-Ps-5.1*, *SPC-Ps-6.1*, and *SPC-Ps-7.1*, respectively, but with contrasting additive effects, reflecting the negative correlation between SPC and SSC.

## 4 Discussion

In the current study, we attempted to understand the genetic basis of SPC in pea using diverse RIL populations using crosses made between high and moderate SPC cultivars. Advances in genomics and the availability of genome sequences have supported the identification of QTLs and candidate genes associated with many complex traits including SPC in grain legumes (Jha et al., 2022). The genetic basis of SPC in many different crop plants is known to be governed by multiple major and minor genes. For example, 241 QTLs associated with SPC have been reported in soybean (soybase.org, accessed 17 November 2023). The complex interaction between these different genes and the environment affects the heritability of SPC. In pea, SPC was demonstrated to have low to moderate heritability (Jermyn, 1977) and was largely influenced by environmental factors such as soil moisture (Tao et al., 2017) and temperature during flowering and pod developmental stages (Karjalainen and Kortet, 1987). The effect of genetic variation and environment and their interaction on the protein content of pea are well known (Daba and Morris, 2021). In the current study, we identified highly significant effects of genetic variation and environment on SPC and GY in two diverse RIL populations. Thus, it is difficult to completely rely on conventional breeding for selection of low heritability traits such as SPC. Like many other crops, in pea as well, a negative correlation between SPC and GY has been reported (Jermyn, 1977; Tar’an et al., 2004). Simultaneously, significant cultivar × environment effects on SPC in pea is also known (Mohammed et al., 2018). We observed a negative correlation between SPC and GY in PR-30 and PR-31 populations, which adds additional challenges for breeding yield and SPC simultaneously. Thus, MAS is desirable to select for high SPC among high yielding lines in a breeding program. The current study was useful to identify the potential targets for MAS of SPC in pea, and also facilitates the exploration and introgression of advantageous natural genetic variability for SPC, which ranges up to ~31% in pea core germplasm (Coyne et al., 2005).

Like many published studies (Daba and Morris, 2021), we observed that the G × E interaction for SPC and GY in PR-30 and PR-31 RIL populations was significant. The correlation between SPC and yield in PR-30 and PR-31 was negative, which is consistent with several previous studies in pea (Jermyn, 1977) and other legume crops (e.g., Obala et al., 2020). The G × E interaction on SPC at the molecular level has been reported in soybean. Hooker et al. (2023) studied the differential gene expression in soybean genotypes with varying levels of SPC grown in different environments and identified that seed protein-related genes, mainly asparaginase and asparagine synthetase, were influenced by the environment.

In the current study, major and minor QTLs associated with SPC, distinguished by their LOD scores, were identified in PR-30 and PR-31. These QTLs are positioned on different linkage groups. Based on sequence-based comparisons of their positions on the reference pea genome sequence (Kreplak et al., 2019), none of these eight QTLs were co-localized. These QTLs were also compared with the three QTLs earlier identified in PR-25 (Zhou et al., 2022), which was also a RIL population derived from a cross between a high SPC and moderate SPC cultivar. The peak of PC-QTL-3 in the PR-25 population overlapped with *SPC-Ps-5.1* in the PR-31 population based on the position of flanking markers on the pea reference genome, which indicates that *SPC-Ps-5.1* is valuable for MAS of SPC. Overall, the diversity of SPC QTLs in mapping populations derived from different cultivars further indicates the complex genetic basis of this trait. The eight QTLs reported are contributed by four moderate or high SPC pea accessions and adds to the list of potential QTLs for MAS of SPC.

Several SPC-associated QTLs have been reported in pea in earlier studies. Gali et al. (2018) identified SPC QTLs in two related RIL populations, PR-02 (Orb × CDC Striker) and PR-07 (Carrera × CDC Striker). Two QTLs positioned on LG1b and LG4a were identified in the PR-02 population. The flanking marker of the QTL on LG4a, Chr4LG4_28114041 (PsC16121p109), is within the range of *SPC-PS-4.1* identified in the PR-30 population. The QTL identified on LG3 and LG7 in the PR-07 population did not match those identified in PR-30 and PR-31. Several SPC QTLs were also detected in other studies involving PR-31 evaluated in French environments (Bourgeois et al., 2011; Klein et al., 2020). These QTLs showed co-locations with *SPC-Ps-3.1, 5.1, 6.1*, and *7.2*.

In a GWAS conducted based on representatives of pea accessions from global pea breeding programs, Gali et al. (2019) identified significant marker–trait associations for SPC. The important markers identified, Chr5LG3_145264443, Chr3LG5_138253621, and Chr3LG5_194530376, did not co-localize with the SPC QTL identified in this study. It must be noted that PR-30 and PR-31 were derived from accessions known for high SPC and are ideal populations for QTL mapping of SPC. Klein et al. (2020) identified several SPC meta-QTLs across the linkage groups. The LOD values of QTLs identified in the current study and the percent phenotypic variance explained by these QTLs are higher than known QTLs, and thus are potential candidates for MAS of SPC.

Eight QTLs associated with GY in PR-30 and PR-31 mapping populations were also identified in this study. These QTLs explained a significant phenotypic variance of GY ranging from 5.0% to 15.4% and are positioned on five chromosomes. The genomic positions of the SPC-associated QTL *SPC-PS-5.1* and the yield-associated QTL *GY-Ps-5.3* in the PR-31 population were co-localized. These QTLs also shared their peak positions and differed by the alleles contributed by the parents in this region. The contribution of the same QTL for either SPC or GY with positive or negative additive effect provides a further validation of the negative correlation between SPC and GY. In addition, co-localization of SPC QTLs with those of PH, DTM, and SSC, with opposite additive effects for SPC and other traits, indicates that simultaneous selection of SPC and other characteristics needs a careful consideration of the trade-offs in breeding for high SPC and high yielding cultivars. It is of notable consideration that four of the five SPC QTLs identified in PR-31 are co-localized with SSC QTLs with opposite additive effects, which is in synchronization with the negative correlation between SSC and SSC. The co-localization of QTLs for SPC and other traits indicate that these traits are controlled by either closely linked genes or the same genes with pleiotropic effects. Obala et al. (2020) made similar observations in pigeonpea that co-localized QTLs of SPC and other yield traits varied in their additive effect values from positive to negative or vice versa. Klein et al. (2020) identified co-localized QTLs for SPC and TSW in pea. The QTLs *SPC-Ps-5.1* and *TSW-Ps-5.2* identified in this study co-localized and the additive effect of both these QTLs was a positive value. The summary of previous and current findings on co-localized QTLs varying in their additive effects substantiate the need for fine mapping of SPC QTLs to breed for SPC in a high-yielding and/or a good agronomic background. We have developed three new mapping populations derived from crosses between CDC Lewochko (Warkentin et al., 2022) and the high SPC parents of PR-25, PR-30, and PR-31, which are CDC Limerick, P0540-91, and Cameor, respectively. Identification of QTL associated with SPC in these new mapping populations is in progress to validate the current QTLs in a common, high yielding genetic background.

The SPC QTLs identified in this study identified the complex genetic architecture of SPC in two different RIL populations. These QTLs, in addition to MAS towards breeding for high SPC, can also provide insight into the genetic basis of SPC in pea at the gene level, helping to elucidate the molecular mechanisms underlying this important trait. Such information through fine mapping of these QTLs facilitates future research on seed protein biosynthesis and develops new approaches to improve the nutritional quality of plant-based protein sources. Overall, the identification of SPC QTLs in PR-30 and PR-31 contributes to improve the nutritional quality of the pea crop and, in that way, contributes to the development of more sustainable and environmentally friendly sources of plant-based protein.

In conclusion, the SPC QTLs identified in this study were contributed for by four pea accessions with high or moderate SPC. These QTLs are potentially important for improving the seed nutritional quality of pea through MAS in breeding programs. The co-localization of two QTLs cautions the careful deployment of MAS for simultaneous selection of high SPC and high yield. Three QTLs *SPC-Ps-4.2*, *SPC-Ps-5.1*, and *SPC-Ps-7.2* contributed by P0540-91, Ballet, and Cameor, respectively, can be used by plant breeders to select the corresponding alleles and develop crop varieties with higher protein content.

## Data availability statement

The original contributions presented in the study are included in the article, further inquiries can be directed to the corresponding author. Any additional raw data supporting the conclusions of this article will be made available by the authors, without undue reservation.

## Author contributions

KG: Conceptualization, Formal analysis, Methodology, Writing – original draft. AJ: Methodology, Writing – review & editing. BT: Conceptualization, Funding acquisition, Resources, Writing – review & editing. JB: Methodology, Writing – review & editing. GAu: Methodology, Writing – review & editing. DB: Methodology, Writing – review & editing. GAr: Methodology, Writing – review & editing. TW: Conceptualization, Funding acquisition, Resources, Supervision, Writing – review & editing.
